# A Syndrome of Variable Allergy, Short Stature, and Fatty Liver

**DOI:** 10.3389/fgene.2021.784135

**Published:** 2022-01-24

**Authors:** Jing Qiao, Yue Chen, Ying Lu, Tiejun Wang, Xiaoli Li, Wei Qin, Aifen Li, Guangquan Chen

**Affiliations:** ^1^ Department of Pediatrics, Shanghai East Hospital, Tongji University School of Medicine, Shanghai, China; ^2^ Department of Clinical Laboratory, Shanghai East Hospital, Tongji University School of Medicine, Shanghai, China; ^3^ Department of Pharmacy, Shanghai East Hospital, Tongji University School of Medicine, Shanghai, China; ^4^ Department of Pediatrics, Jian Hospital of Shanghai East Hospital, Jian, China; ^5^ Fetal Medicine Unit & Prenatal Diagnosis Center, Shanghai First Maternity and Infant Hospital, Tongji University, Shanghai, China

**Keywords:** *SLC22A18*, imprinted gene, allergy, short stature, fatty liver

## Abstract

*SLC22A18* (solute carrier family 22 member 18) is an imprinted gene, but its role in growth and development is not clear. In the present study, we recorded the clinical information of six male patients of six unrelated families. Real-time quantitative PCR, Sanger sequencing, and DNA methylation sequencing were performed in these patients. The results suggested that the patients with the clinical characteristics of allergic allergy, short stature, and fatty liver had a lower expression of *SLC22A18*. One novel variant (chr11: 2899732 delA) with clinical significance was found in the core promoter region of the patients. Overall, this study found a syndrome associated with *SLC22A18*.

## Introduction

The prevalence of allergic diseases has been increasing worldwide over the past 60 years, affecting about 30% of the global population (Palomares et al., 2017). A phenomenon known as “allergic march” had been firstly described by Fouchard in 1973. It is a process from infant eczema to food allergy, asthma, and rhinitis resulting in poor quality of life in childhood (Sohi and Warner, 2008).

In clinical practice, a common triad, including variable allergies, short stature, and fatty liver, has not been reported as a syndrome up to now. Previous studies on allergic diseases mainly focused on the immunogenic origin of allergic diseases, the clinical significance of “health hypothesis,” and the impact of maternal and infant nutrition on allergic epidemics and paid little attention to the role of human imprinted genes.

*SLC22A18* is an imprinted gene, which is involved in tumor suppression and lipid accumulation. Diseases associated with *SLC22A18* include lung cancer and breast cancer (Dao et al., 1998; Peters, 2014; Ito et al., 2019), but its role in childhood diseases is not clear.

In this study, we describe six male patients from six unrelated families with a triad symptom of progressive postnatal slow growth, allergies, and fatty liver. After real-time quantitate PCR (RT-qPCR), Sanger sequencing, and DNA methylation sequencing analysis, we showed that all the patients had a lower expression of *SLC22A18* that resulted from abnormal methylation-hampered promoter function. These cases and analysis indicate a syndrome associated with *SLC22A18*.

## Methods

### Cases

From Nov 2013 to Aug 2020, six male patients from six unrelated families who were admitted to the pediatric endocrinology clinic presented a triad symptom of progressive postnatal slow growth, allergies, and fatty liver. After reviewing the patients’ family history, we found that the patients’ grandfathers or fathers also had similar growth experience compared with the patients. Subsequently, the medical history, physical findings, and the results of hematology, biochemistry, radiology, type B ultrasonic test, and molecular biology tests were studied. B-ultrasound examination found that the patients’ liver had infiltration, suggesting that the patient had fatty liver. All laboratory procedures for clinical samples have been reported in advance. Blood, feces, and urine samples were taken; plasma was separated in the EDTA bottles; and serum was separated in the clotting blood bottles. This study was approved by the Institutional Review Board and Ethics Committee. Written consent from all patients was collected.

### Real-Time Quantitate PCR

Whole blood of 10 patients (IDs: 19010101, 19010102, 19010104, 190101010, 19010111, LXY, DTY, LC, OYZY, and GJX) and 10 healthy controls were first processed with Red cell lysis buffer (Sangon Biotech, Shanghai, China) and then treated with TRIzol (Invitrogen, Carlsbad, CA, USA) to extract total RNA. Reverse transcription was performed with HiScript First Strand cDNA Synthesis Kit (Vazyme, Nanjing, China) to obtain cDNAs. Then qPCR was performed on Bio-Rad CFX96 (Bio-Rad Laboratories Inc., Hercules, CA, USA) with AceQ qPCR SYBR Green Master Mix (without ROX) (Vazyme, Nanjing, China) according to the manufacturers’ protocols. *ACTB* gene was used as the reference, and the primer sequences are listed in Supplementary Table S1. Cycling conditions were as follows: 95°C for 5 min, followed by 40 cycles of 95°C for 10 s and 60°C for 25 s.

### DNA Methylation Sequencing and Data Analysis

Genomic DNA was extracted from whole blood using TIANamp Blood DNA Kit (Tiangen Biotech, Beijing, China), which was further treated with EpiTect Fast DNA Bisulfite Kit (QIAGEN, Hilden, Germany) for bisulfite conversion. The converted DNA was PCR-amplified with primer sequences designed to cover the CpGs in two promoter regions denoted as “Promoter 1” and “Promoter 2” (Supplementary Table S2): each region included near 1,000 bp centering around the transcription start sites (TSSs) of SLC22A18; the TSS annotation was based on RefSeq release 109. The PCR products were gel- and column-purified and used for DNA library preparation. The library was prepared with KAPA HTP Library Preparation Kit (KAPA Biosystems, Wilmington, MA, USA) according to the manufacturer’s protocol. The library was further amplified for 10 cycles, which was then subjected to deep sequencing on the Illumina HiSeq platform with 2 × 150 as the sequencing mode.

Raw reads were filtered to obtain high-quality clean reads by removing sequencing adapters and low-quality reads using Trim Galore (v0.5.0) with parameters--paired--rrbs--illumina--fastqc (https://github.com/FelixKrueger/TrimGalore) (FastQC, 2010; Martin, 2011). The clean reads were mapped to human genome (hg38) using the Bismark (v0.7.0) software (Krueger and Andrews, 2011). The methylation percentages for the CpG sites were calculated by the Bismark methylation extractor script from Bismark. Differentially methylated CpGs (DMCs) were identified using methylKit with the *q*-value cutoff set to 0.01 (Akalin et al., 2012). Differentially methylated regions (DMRs) between patients and healthy controls were identified within the two promoter regions using methylKit, which had a *q*-value of less than 0.01 and at least one DMC inside.

### Sanger Sequencing

Genomic DNA was extracted from the whole blood of nine patients (IDs: 16, 17, 20, 170609, 17071201, 19010101, 19010106, 19010109, and TANG) with the same method described above. Two pairs of PCR primers were used to amplify the promoter regions of SLC22A18 (Supplementary Table S3), yielding close to 500 bp flanking the TSSs. PCRs were performed in a 50-μl reaction containing 10 μM of each primer, 100 ng of genomic DNA, and 25 μl of 2xFtaq PCR MasterMix (Zoman Biotechnology, Beijing, China). Cycling conditions were as follows: 95°C for 5 min, followed by 40 cycles of 95°C for 15 s, 60°C for 15 s, and 72°C for 50 s. The PCR products were gel- and column-purified and then sequenced with ABI 3730XL (Applied Biosystems, Foster City, CA, USA).

## Results

### Clinical Features of the Six Cases

**FIGURE 1:**
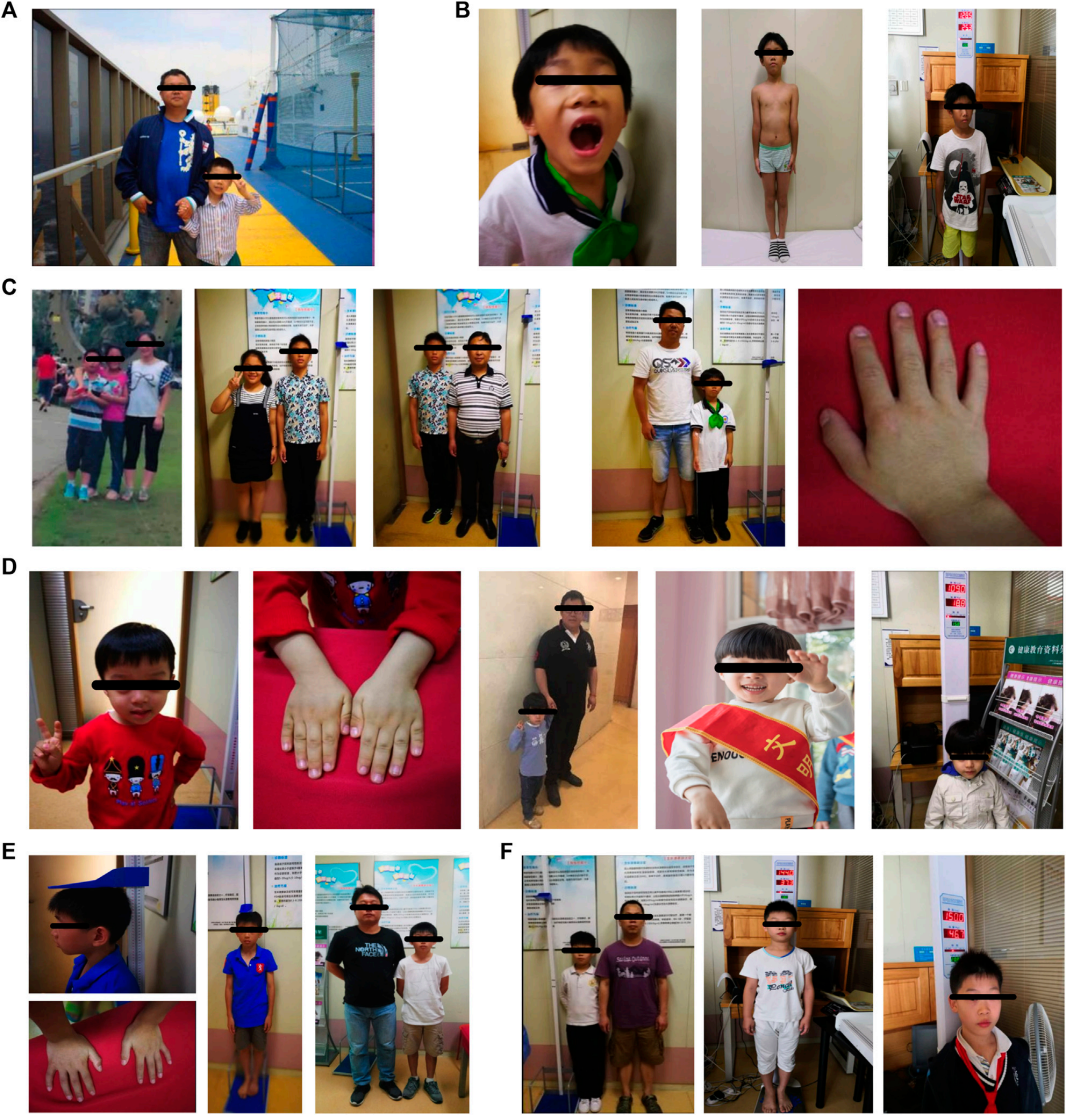
Clinical features of the six cases’ profiling.

The patient in case 1 was short and light, and his father had marked central obesity (Figure 1A). In case 2, the patient had short stature, was lightweight, and has small hands and teethed at a normal age, and his father had marked central obesity (Figure 1B). The patient of case 3 is the brother of boy–girl twins. He was thinner and shorter than his twin sister before 12 years old (left picture). However, after 1.5 years of recombinant human growth hormone (rhGH) treatment, he is 9 cm taller than his twin sister (middle picture) now. His father suffered from central obesity (Figure 1C). The patient of case 4 was of short stature and lightweight and has small hands. He teethed at a normal age, and his father had marked central obesity (Figure 1D). The patient of case 5 has short stature without spinal scoliosis, was lightweight, and has small hands, and his father had marked central obesity (Figure 1E). The patient in case 6 was short, with a body mass index (BMI) of 18.0, and had fatty liver, and his father had central obesity (Figure 1F).

**Case 1** The initial dose of rhGH is 2-3 IU/d over a period of 18 months and he received total 1248 IU of rhGH. During this period, his height and weight increased by 12.9 cm (to 121.0 cm) and 6.8 kg (to 23.0 kg). Then, he suspended injection rhGH since his height became normal compared to his peers, after approximately 10 months, his height and weight was still 121.0 cm and 23.0 kg, without any appropriate increase. Then started using rhGH, 4 IU/d, 6 days/week. Up to date, when he was 9y9m, both of his height and weight were normal (137.9 cm, 30.3 kg). At this time, the dose of rhGH is 5 IU/d, 6 days/week.

**Case 2** When he was 3y10m years old, the injections were started and lasted for approximately 1.5 years, by a frequency of rhGH 2 IU/d, 6 days/week. Afterwards, he had a remarkable height increase which is from 95.6 cm to 112.5 cm. Sometimes he suspended for 3 months because the great treatment effects. Last revisited at Jan, 1, 2019, his height was 128.5 cm (normal), and weight was 25.3 kg.

**Case 3** When he was 12.3 years old, given him VitD3 supplements and treated with rhGH 6 IU/d. The tratments were continued for approximately 3 years (from Nov. 2015 to July. 2018). Last revisited was at 14.8 years old, and his height was 171.0 cm and weight was 67.5 kg.

**Case 4** To data, his height was 101.5 cm (-3SD) and weight was 17.0 kg (-1SD), when he was five years old. Injection of rhGH with 2 IU/d was recommended. After 4 months, his height was 109.0 cm and weight was 17.0 kg, which means effective treatments, however, he still had cough and lean body mass.

**Case 5** He received 5 IU/d rhGH, over a period of six months, with height increased from 137.5 cm to 145.0 cm, and weight was 34.7 kg (normal height increases by 3.0 cm in six months). Treatment was interrupted for the following six months, resulting in no further growth. We suggested the patient visiting E.N.T. department for his sleep problems and traditional Chinese medicine for his poor of appetite. Now, he is 158.0 cm and 46.0 kg, and his appetite has improved.

**Case 6** When he was 11.5 years old, he started using rhGH (4 IU/d). Well, at that time of Aug 23, 2018 his height was 144.0 cm and weight was 37.3 kg. Suggested him using rhGH (5 IU/d) and the treatment effects were very effective for height.

All cases were male with normal birth height and weight. When these patients visited our department, they had low body weight and slow growth, similar to idiopathic short stature (ISS).

**FIGURE 2:**
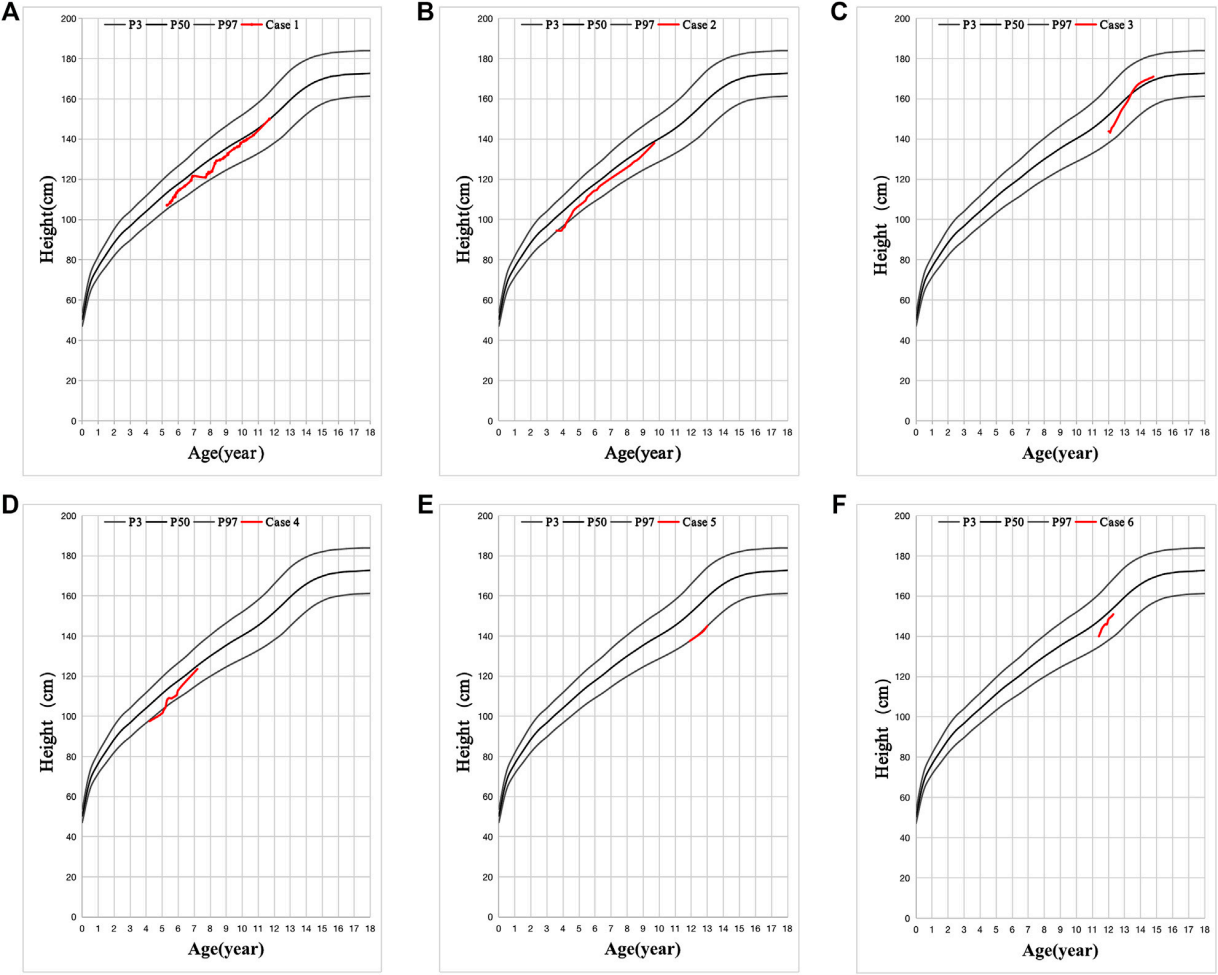
Six cases with favorable effect to recombinant human growth hormone (rhGH) treatment. **(A-F)** is the height changes in six cases. The black line is the standard height curve from top to bottom is 97, 50, and 3%, respectively. Height below the 3rd percentile is considered as Short Stature. The red line is the height changes of these 6 patients after VitD3 and rhGH treatment.

All cases had hypertrophy of adenoid and tract allergy of the upper respiratory tract. Besides, we found that the grandfathers or fathers of the six patients also showed nearly the same combination of short stature, allergic march, and fatty livers during their puberty. Growth hormone deficiency (GHD) was found in five cases, except in case 5. Patients had decreased vitamin D_3_ (VitD_3_) and insulin-like growth factor-1 (IGF-1) and increased free fatty acid (FFA). MRI of the pituitary showed no abnormalities, and the intelligence and sexual development of these patients were normal (Table 1). These patients were diagnosed with GHD or ISS, along with other diagnoses such as an allergic reaction of the upper respiratory tract, asthma, and fatty liver (Table 1). After VitD_3_ supplementation and rhGH treatment, their height increased by >7 cm/year (Figure 2).

**TABLE 1:** Summary of main clinical features and laboratory results of six cases with SLC22A18-associated syndrome.

		Case 1	Case 2	Case 3	Case 4	Case 5	Case 6
Age (at first examination) (year–month)		5 years + 3 months	3 years + 7 months	12 years	4 years + 2 months	11 years + 11 months	11 years + 2 months
Gender		Male	Male	Male	Male	Male	Male
Patient’s height (cm)		107.1	94.5	143.8	97.5	137.5	140
Patient’s weight (kg)		16.3	13.4	44.5	14.4	29.5	34.7
Patient’s BMI (kg/m^2^)		14.17	15.01	21.52	15.15	15.6	17.7
Birth’ history	Gestation (weeks)	40w+2	40w−4	40w−3	39w+6	41 + 1	40w+4
	Birth height (cm)	50	49	50	50	45	50
	Birth weight (kg)	3.4	3.25	3.1	3.3	3.3	3.8
Father’s height (cm)		160	167	169.4	169.6	167.5	161.5
Father’s weight (kg)		74.3	70	91.7	108	83	84.5
Father’s BMI (kg/m^2^)		29.02	25.1	31.96	37.55	29.58	32.4
Father’s chronic medical illness		Fatty liver central obesity	Fatty liver	Fatty liver central obesity chronic	Fatty liver central obesity chronic	Fatty liver central obesity	Fatty liver central obesity chronic
Mother’s height (cm)		152.7	155	152.8	154	154	158
Mother’s weight (kg)		47.2	47	53.9	54	53	50
Mother’s BMI (kg/m^2^)		20.2	19.6	23.1	22.8	22.3	20
Symptoms and signs							
Low growth velocity		+	+	+	+	+	+
Short stature		+	+	+	+	+	+
low weight (low body mass)		+	+	Normal	+	+	Normal
Snoring		+	+	+	+	+	+
Adenoid hypertrophy		+	+	+	+	+	+
Allergic rhinitis		+	+	+	+	+	−
Asthma		+	−	−	+	−	+
IGF-1 (μg/ml)		354 (↓)	139	391 (↓)	240	178 (↓)	113 (↓)
IGFBP-3 (μg/ml)		5	4.79	5.7	4.1	4.8	4.4
VitD_3_ (ng/ml) (normal range ≥30)		28.3 (↓)	35.2	23.9 (↓)	20.9 (↓)	14.9 (↓)	22.9 (↓)
Peak of GH (μg/L) (normal range >10)		8.4 (↓)	3.98 (↓)	2.4 (↓)	7.61 (↓)	19.3 (↑)	6.74 (↓)
Hemoglobin (normal range 120.0–140.0 g/L)		126	118 (↓)	152	127	145	112 (↓)
Neutrophils (%) (normal range 40.0–75.0)		49	32.1	74.5	54.6	59	63.7
Lymphocytes (%) (normal range 20.0–50.0)		36.5	54.9	13.5	30	32.5	25.9
Monocytes (%) (normal range 1.0–8.0)		8.4 (↑)	8.4 (↑)	8.4 (↑)	12.6 (↑)	4.7	10.1 (↑)
Eosinophils (%) (normal range 0.4–8.0)		5.2	5.2	3.1	2.5	2.9	0.0 (↓)
Determination of serum allergens		Pollen	Mite		Pollen, egg	Pollen	Mite, egg
TG (mmol/L) (normal range 0–5.2)		4.2	4.5	4.2	4.6	4.2	4.26
TC (mmol/L) (normal range 0–2.26)		0.7	0.6	0.9	1.25	0.5	1.46
HDL-C (mmol/L) (normal range ≥1.04)		2.2	1.8	1.5	1.3	1.9	1.72
LDL-C (mmol/L) (normal range ≤3.34)		1.9	2.6	2.6	3.04	2.37	2.4
APOA (g/L) (normal range 1.04–2.02)		1.76	1.97	1.5	1.3	1.54	1.68
APOB (g/L) (normal range 0.66–1.33)		0.5 (↓)	0.8	0.8	0.9	0.69	0.76
APOE (mg/L) (normal range 27–45)		55.9 (↑)	35.3	50.4 (↑)	62.0 (↑)	39	54.0 (↑)
FFAs (mmol/L) (normal range 0.1–0.6)		0.95 (↑)	1.15 (↑)	0.86 (↑)	0.86 (↑)	0.67 (↑)	0.94 (↑)
Glucose (mmo1/L) (normal range 4.11–6.05)		4.8	4.5	5.4	4.6	4.3	4.9
HbA1c (%) (normal range 4–6)		4	4.1	5	4.3	4.2	5
Insulin (%) (normal range 2.6–24.9)		3.7	4.4	5.5	3.7	4.5	5.1
Liver and renal function		Normal	Normal	Normal	Normal	Normal	Normal
Fatty liver (ultrasonic B)		−	−	+	−	−	+
Pituitary MRI		Normal	Normal	Normal	Normal	Normal	Normal
Chronological age (CA) (year)		5.3 years	7 years	14 years	4 years	12 years	10.5 years
Bone age (BA) (year)		2.5 years	4.5 years	13.5 years	1.5 years+	9 years−	8 years
X-ray of BA and CA		BA < CA	BA < CA	BA = CA	BA < CA	BA < CA	BA < CA
Diagnosis							
Endocrinologist		GHD	GHD	Short stature	GHD	ISS	GHD
						Puberty state	Puberty state
Otolaryngologist		Adenoid	Adenoid	Adenoid	Adenoid	Adenoid	Adenoid
Pulmonary physicians					Asthma		
Gastroenterology				Fatty liver			Fatty liver
Treatment	rhGH	+	+	+	+	+	+
	Adenoidectomy	+	+	−	+	−	+
	Antiallergic therapy	+	−	−	+	−	+
	Vitamin D_3_	+	+	+	+	+	+

Note. BMI, body mass index; TG, triglyceride; TC, total cholesterol; HDL-C, high-density lipoprotein cholesterol; LDL-C, low-density lipoprotein cholesterol; FFA, free fatty acid; HbA1c, glycated hemoglobin; GHD, growth hormone deficiency; rhGH, recombinant human growth hormone.

### Quantification of *SLC22A18* Expression Level

**FIGURE 3:**
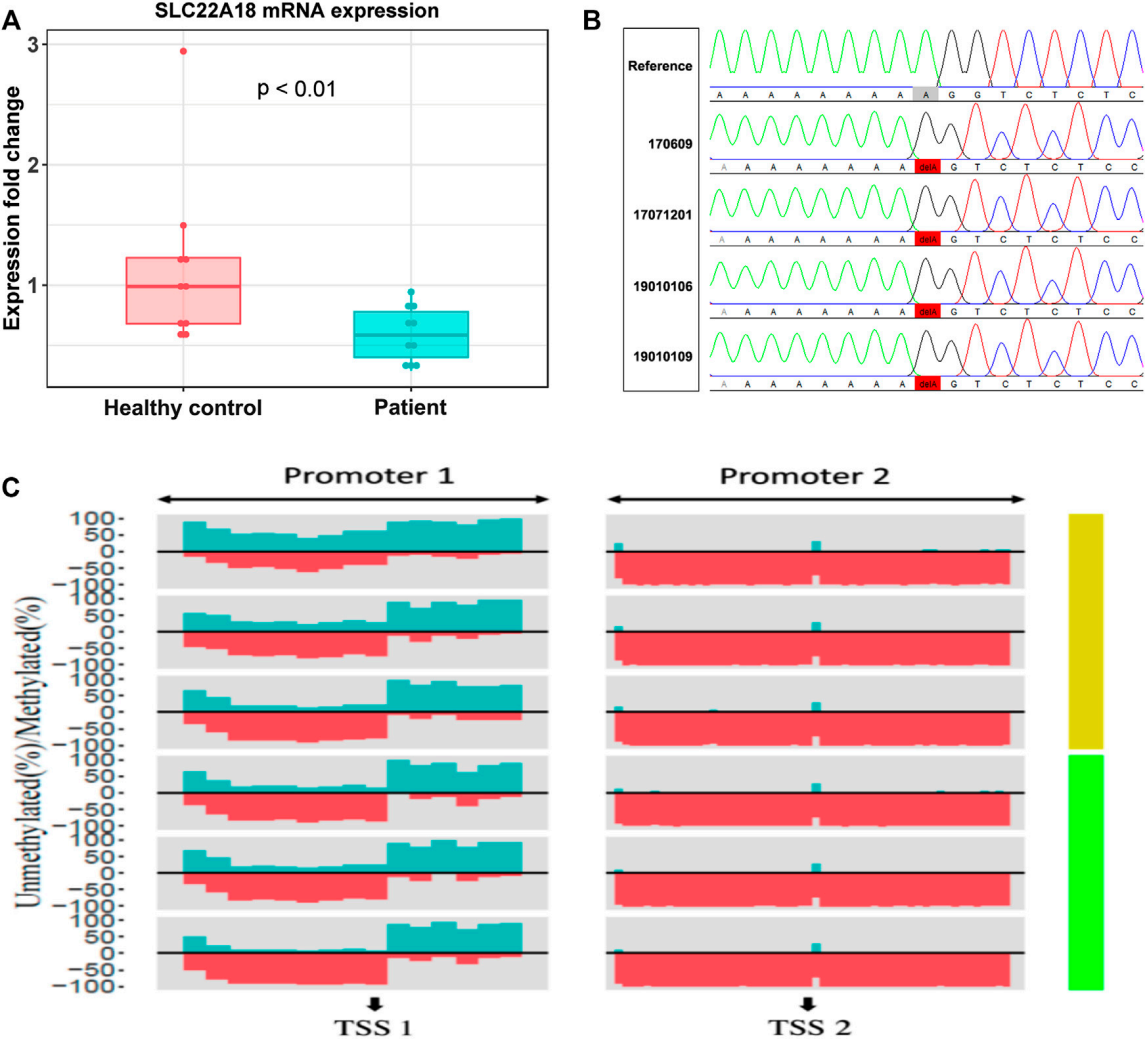
The focal point of a patrilineal imprinted gene *SLC22A18*. **(A)** Comparison of SLC22A18 expression levels between patients and healthy controls. There are 10 individuals in each group, and the expression levels were measured by RT-qPCR, and fold changes were calculated with the healthy controls as the reference. The *p*-value indicated in the plot was based on a two-tailed Student’s t-test in the R language. **(B)** The left panel shows the IDs of patients with this variant, and the reference sequence is on the top. The solid red box highlights the variant where an “A” has been deleted. The visualization of Sanger sequencing results was done by novoSNP. **(C)** The percentage of methylated and methylated CpG sites was represented by dark green and red, respectively. Patients and healthy controls were represented by light green and yellow bars, respectively, on the right side. “TSS1” is located at position 2, 899, 721, “+” strand, chromosome 11; “TSS2” is located at position 2, 902, 282, “+” strand, chromosome 11.

RT-qPCR was used to measure the expression level of *SLC22A18* in both patients and healthy controls. A significant difference in expression was detected between patients and healthy controls (Figure 3A). On average, the expression of *SLC22A18* in the healthy control group was 1.82 times higher than that in the patient group.

### Methylation Analysis and Variant Detection in Promoter Regions of *SLC22A18*

Aberrantly, DNA methylation is one of the most possible reasons to drive the expression of *SLC22A18* to become abnormal. Here, we quantified the promoter methylation levels of three patients (IDs: 19010102, 19010110, and LC) and three healthy controls by deep sequencing. Deep sequencing generates 2.7 to 4.8 million reads for each individual, leading to an ultra-high coverage (>50,000× on average) for two specific promoter regions (denoted as “Promoter 1” and “Promoter 2”), each of which included nearly 1,000 bp centering around the TSSs of *SLC22A18* (Supplementary Table S2). Using methylKit, DMRs were identified in Promoter 1: 1) the core promoter was the most differentially methylated between patients and healthy controls with 21% methylation percentage change (*p*-value <0.001); 2) the upstream 500 bp of the TSS had a marked elevated methylation percentage (15%, *p*-value < 0.001); and 3) the downstream 500 bp of the TSS had a relatively small increase in methylation (6% change, *p*-value <0.001). On the contrary, the majority of CpG sites in Promoter 2 were nearly 100% methylated, and thus no noted change in methylation level was observed in Promoter 2 (Figure 3C). To identify variants that possibly lead to the low expression of *SLC22A18* in patients, Sanger sequencing was performed for the two promoter regions described above. Genomic DNA was extracted from the whole blood of nine patients (IDs: 16, 17, 20, 170,609, 17,071,201, 19010101, 19010106, 19010109, and TANG), yielding close to 500 bp flanking the TSSs that were subjected to Sanger sequencing. The sequencing results were aligned to the human reference genome (GRCh38) and visualized by novoSNP (Weckx et al., 2005). A total of seven variants were identified and, as expected, most of them (6) could be found in dbSNP (i.e., rs365605, rs5789280, rs538924456, rs397933484, rs366696, and rs367035) without clinical significance (Sherry et al., 2001; Landrum et al., 2014). Only one variant (chr11: 2899732 delA), located in the core promoter region, has not been reported before (Figure 3B). To further check if this novel variant could be related to the expression of *SLC22A18*, the 100-bp DNA sequence flanking this variant was submitted to JASPAR database (https://jaspar.genereg.net/) to detect possible binding sites of transcription factors (Khan et al., 2018). To our surprise, 11 transcription factors (MEF2C, ZNF384, SOX15, LM140, SOX15, SOX10, RORC, RORA, RORB, NR4A1, NR2F2, and NR4A2) could bind to the regions containing the novel variant under default parameters.

## Discussion

Individual children often have multisystem diseases. The clinician needs to be aware of these to ensure that the child is not misdiagnosed. In the past, short stature and obesity in children with allergic diseases are usually considered to be side effects of glucocorticoid drugs. But now, our study generated evidences showing that short stature and central obesity are not related to the use of glucocorticoid drugs. Therefore, we need a new theory to explain the pathological mechanisms of short stature and/or obesity with allergies in children. In this report, the triad of allergic march, short stature, and fatty liver is associated with a patrilineal imprinted gene *SLC22A18*. It should not simply be considered a side effect of glucocorticoid.

There is a progression, and the individual child can suffer from one symptom to another. The most common is normal height and weight at birth. In early childhood, allergies, reduced growth rate, short stature, and abnormal fat metabolism [high FFA and high apolipoprotein E (ApoE)] gradually appear. Around adolescence, weight gain develops into central obesity and fatty liver. And allergic symptoms almost always appear after birth to the age of 7. Other symptoms of itching, sneezing, loss of sleep, coughing, etc., are present. In our study, there is no obvious abnormality in the appearance of our cases, and there is no sexual developmental delay and intellectual disability in any of these patients. The group of six patients could not be classified into the above syndromes.

Several syndromes involving short stature are associated with a number of imprinted genes, such as Prader–Willi syndrome (PWS), Beckwith–Wiedemann syndrome (BWS), and Silver–Russell syndrome (SRS). PWS is characterized by short stature and obesity, somewhat similar in our patients. However, inborn muscular hypotonia, imbecility, and cryptorchidism, and/or micropenis are not observed in our cases (Cassidy et al., 2012). BWS features varying degrees of symptoms like overgrowth, macrosomia, macroglossia, hemihypertrophy, and asymmetric facial features (Frédéric et al., 2018). SRS is characterized by severe intrauterine and postnatal growth retardation, feeding difficulties, short stature, triangular face, low ears, and bending of the fifth finger (Spiteri et al., 2017). The group of six patients could not be classified into the above syndromes.

Short stature is the most confusing common feature in the cases of this study. During follow-up, six cases showed a favorable effect of rhGH treatment. All patients grew faster and taller, but it was almost impossible to avoid allergies, consistently elevated plasma levels of IgE, FFA, and fatty liver formation. The molecular mechanism is still unknown, and rare evidence can be found by existing researches.

*SLC22A18*, located in the 11p15.5 domain, is an important tumor-suppressor gene region. Alterations in this region have been associated with the BWS, Wilms tumor, and lung, ovarian, and breast cancers. Lee et al. found mutations in SLC22A18 in kidney and lung cancers. By checking the genotypes and phenotypes of the family members, the author speculated that SLC22A18 is a tumor suppressor gene in the adult lung and an imprinted tumor suppressor gene in the fetal kidney (Lee et al., 1998). Recent studies revealed a novel link between *SLC22A18* and fat accumulation, and we speculated that paternal *SLC22A18* gene may be involved in the occurrence of this “triad” syndrome. Previous reports found that the liver expresses *SLC22A18* at the mRNA and protein levels (Dao et al., 1998). Suppression of *SLC22A18* promotes lipid accumulation in the liver by reducing lipophagy (Jing and Gotoda, 2012; Yamamoto et al., 2013; Shingo et al., 2019). Dysregulation of lipid metabolism in the liver is a marker of nonalcoholic fatty liver disease (NAFLD), characterized by excessive accumulation of fat in the liver. These findings may explain the development of fatty liver in our cases.

Given these findings and the biological importance of *SLC22A18*, the DNA sequence and the RNA levels of *SLC22A18* were investigated among these patients. It was found that high methylation and low expression of SLC22A18 could relate to the occurrence of a triad of slow growth, allergies, and fatty liver in these patients during their growth and development. Therefore, it is suggested that *SLC22A18* is a possible gene that plays an important role in the pathogenesis of this syndrome.

In summary, this study found that the triad, variable allergy, short stature, and fatty liver, is associated with the lower mRNA expression levels of SLC22A18, deleted “A” in SLC22A18 core promoter, and the high methylation levels in my cases. All of that possibly affect the normal transcription of SLC22A18, meanwhile resulting in IGF-1 low activity and involvement of high FFA in metabolic inflammation. Therefore, our study raises the clinical need for the naming of *SLC22A18* syndrome. Last but not least, additional case samples are needed to reinforce our hypothesis; and further researches of *SLC22A18*, such as epigenetics or functional genetic experiments of an animal model, will reveal the molecular mechanisms and etiology.

## Data Availability

The Data Availability statement is corrected to “The datasets presented in this study can be found in online repositories. The names of the repository/repositories and accession number(s) can be found below: https://www.ebi.ac.uk/arrayexpress/, E-MTAB-9579.
