# Supplementary material for: A Syndrome of Variable Allergy, Short Stature, and Fatty Liver
Source: Front Genet. 2022 Jan 24;12:784135. doi: 10.3389/fgene.2021.784135 (PMC8819686; doi:10.3389/fgene.2021.784135)
Supplement: Supplementary file 1 [file Table1.DOCX]

**Table S1. The primer sequences for ATCB and SLC22A18 in RT-qPCR**

| **Gene** | **Forward sequence (5'-3')** | **Reverse sequence (5'-3')** | **PCR Products (bp)** |
| --- | --- | --- | --- |
| ACTB | ACTGGGACGACATGGAGAAAA | TGGCTGGGGTGTTGAAGG | 162 |
| SLC22A18 | ATGTCCTTCTTCGGGCTCC | ACCTCCTCCGAGAAGTGGC | 80 |

**Table S2 The primer sequences for amplifying bisulfite-converted DNA**

The letter “R” stands for A or G and “Y” for C or T.

| **Promoter** | **Forward sequence (5'-3')** | **Reverse sequence (5'-3')** | **PCR Products (bp)** |
| --- | --- | --- | --- |
| 1 | TTTTTTTTAGTGGTTTAGTTTTTTGAATAT | ACAAATAAAACCCTAACACCCCTA | 357 |
| 1 | AGAATTAGGAAGTTTTTTTATTTATATTGA | AACTTAAAAAAAATACAATCAAACC | 477 |
| 2 | TTTTTTTTAGGTTTAAAGTGGATAT | CRCTCCAAAATAACCTAAACACC | 350 |
| 2 | YGGTGTTTAGGTTATTTTGGAG | ACTCCCCAATAACTCTACCTTACCT | 447 |

| **Promoter** | **Forward sequence (5'-3')** | **Reverse sequence (5'-3')** | **PCR Products (bp)** |
| --- | --- | --- | --- |
| 1 | ACTCACTTTCTGCCCCGTCAC | CCCAGATCCTCTGCCAAGC | 979 |
| 2 | CCATTCCTCCGCTTTTCTC | GTGGCCCTGTCCCCAACA | 987 |

**Table S3. The primer sequences for PCR amplification**
