# Supplementary material for: A Syndrome of Variable Allergy, Short Stature, and Fatty Liver
Source: Front Genet. 2022 Jan 24;12:784135. doi: 10.3389/fgene.2021.784135 (PMC8819686; doi:10.3389/fgene.2021.784135)
Supplement: Supplementary file 2 [file DataSheet1.docx]

**Supplementary**

Case 1. J.W. male, age 5 years and 3 months, was first seen by me on November 6, 2013. He was skinny look, walked, talked and teethed at normal age. He was born on July 17, 2008. And showed underdevelopment of the idiopathic short stature (ISS) type. There was a low growth velocity with low weight, the BMI was 14.7kg/m2 at early school age. His height velocity was below -2 SD, and body weight was -3SD of mean for chronological age. He is susceptible to cold and suffered from allergic rhinitis in preschool. And he had done adenoidectomy at Shanghai Children's Medical Center on Mar. 16, 2018. When he was 5years and ten months old, injections of recombinant human growth hormone (rhGH), was begun, six times a week from 2IU to 3IU, and over a period of 18 months he received 1248 IU of this substance. To date, when he was 9Y9M, both of his height 137.9cm and his weight 30.3kg were normal on Jun. 26, 2018. At this time, he uses rhGH, 5IU/qd, 6times/week, the height to get a better effect, there was no slightly increased appetite and MBI, however there had been no any change in lipid metabolism, his blood of NEFA and APOE still showed a very increased.

Case2. XH.Z., male, age 3 years and 7months (born on April 16, 2010) was first seen by me on February 11, 2014. He always no hunger and appetite. He was slow growth and suffered from allergic cough. His height velocity was less than 4cm/year at age for 3-5year. The patient showed thin and small underdevelopment of growth hormone deficiency (GHD) type. His height was 94.5cm (-3SD), and weight 13.4kg (-3SD). When he was 3 years and 10 months old, he did GHmax of GH stimulation test showed 3.98μg/l was marked lower than normal. He had bone age test on Apr.15, 2017 however, his bone age was 3.5 years behind normal children of same age. And CT of head examination showed no abnormal was found. rhGH, 2IU/ 6 times weekly, was prescribed, and injections were continued for approximately 1.5 years with outstanding increased in his height from 95.6 to 112.5cm. Sometimes he suspended for 3 months because the treatment’s effect is good enough. Last revisit Jan.1,2019 (8.8y) His height was 128.5cm (normal) and weight was 25.3kg.

Case3. MH.G, male, age 12 years, born on Aug.1, 2003, was first seen by me with his twin sister on July 23, 2015. They walked, talked, and teethed at the normal age. He is half a head shorter than his twin sister, his twin sister looks his old sister. His stature has always been less than that of his twin sister of the same day. There was a less than 4cm/year of height velocity at age for school age. The patient showed underdevelopment of the idiopathic short stature (ISS) type. His height was 143.7cm (-2SD), and weight 44.5kg (+1SD). He has no asphyxia at birth, His birth height was 50cm and birth weight was 3.1kg (his twin sister was 2.8kg/48cm). He studies very well, but suffered from asthma on his preschool age. The blood serology and chemistry of sex hormone level was increased means his puberty has begun. At this time, the patient was deficient in vitamin, his VitD_3_ was 23.8ng/ml, and no reason to explain of his high APOE and NEFA of lipid metabolism, his B ultrasonic also showed fatty liver, and his peak of GH showed 2.4ug/l was very lower than normal. Besides, His sex started developing without good growth velocity of height, therefore, he was given VitD_3_ supplements and treated with rhGH, 6IU, 7 times weekly, injections were continued for approximately 3 years (from Nov. 2015 to July.2018). Last revisit he was 14.8years, and his height was 171cm and his weight was 67.5kg.

Case 4. JK.Z., male, age 1-year and 2-month, was first seen by me on June 15, 2014. He coughed whoop and aloud cough without fever, at that time he was diagnosed with asthmatic bronchitis and was promptly treated. Since then, he has suffered repeated bouts of wheezing, and his respiratory doctor has evaluated and monitor regularly in order to adjust the treatment plan. Well, he could get regular anti-asthma treatment. His illness is well under control. After 3 years, he seen me again. There was a low growth velocity below -3SD of mean, and he has always been less than that of his playmates of the same years after he was born. In addition, He also suffered from adenoids hypertrophy, when he was more 5years. He was found snoring with his mouth open when sleeping for 3 months. Then he visited Xinhua Hospital of Medical School of Shang Hai Jiao Tong University, Paranasal sinus CBCT found the patient has adenoids hypertrophy and partial airway compressions. Thus, he done endoscopic adenoid aspiration and radiofrequency ablation of bilateral tonsils and radiofrequency ablation of bilateral inferior turbinate at Xinhua Hospital of Medical School of Shang Hai Jiao Tong University on Sep. 2018. To data, his height was 101.5cm(-3SD) and weight was 17kg(-1SD), when he was five years. Suggested him to use rhGH, 2IU, 7 times a week. After 8 months, his height was 113cm and his weight was 21.2kg, that showed good effect for the treatment of height, however, he still easy cough.

Case 5. C.C.，male，age 11-year and 11-month (born on August.31,2005.), was first seen by me on July10, 2017. He walked, talked, and teethed at the normal age. His has always been less than that of his playmates of the same years. The patient showed low weight underdevelopment of the ISS type. His height was 137.5cm (-2SD), and weight 29.5kg (-2SD). There was a marked low growth velocity after he birth. He has always had a poor appetite and eats very little, except meat. He has also bad sleep. He suffers from allergic rhinitis since his 5 years old and he allergies to air conditioning since his 10 years old. His lower left leg got bony crack about 10 years ago，treated by plaster fixation. His birth height was 48cm and birth weight was3.3kg. He has no asphyxia at birth. Mental is normal. His VitD_3_ was decreased, only 14.90 ng/ml, and GHmax of GH stimulation test showed 19.6μg/l was higher than normal. When he was 11-year and 11-month old, He did bone age examinations, however, his BA was 3 years behind CA. And MRI of his head, skull and sella, and pituitary gland indicated normal development. He received rhGH, 5IU/qd. Subcutaneously, over a period of six months, with an increase in her height of 145.0cm, and weight was 34.7 kg (theoretical normal increment 3cm). Further treatment during the following 6 months, interspersed with rest periods, resulted in no further growth. Suggested the patient visit E.N.T. department for his sleep is not good enough. As well as to consult traditional Chinese medicine for the patient poor of appetite.

Case 6. JR.L, male, age11-year and 3-month, was first seen by me on May 7, 2018. His stature has always been less than that of his playmates of the same years after he born. His height was 140cm (-2SD), and weight 34.6kg (-1SD) that showed small underdevelopment of ISS type. There was a low growth velocity below 3cm/year. He has a good appetite and prefers eating meat and has no bad sleep. He has allergic constitution and suffers from allergic rhinitis and profuse sweating. His birth height was 50cm and birth weight was 3.8kg. There was a bit evidence of secondary sex characters. And the blood serology and chemistry showed his NEFA is 0.67 mmol/L of lipid metabolism increased, and his APOB is 0.56 g/l decreased. His of sex hormone level showed LH is 1.65 mIU/ml, FSH is 4.8, and PRL is 11.8mIU/ml, that all of those increased it means he has entered the state of sexual development. More, VitD_3_ of Vitamin metabolism was decreased, and peak of GH showed 6.74 μg/l was lower than normal. In addition, He had first time to bone age test on May 26,2018. However, his BA was 3 years behind normal children of same age. And MRI of his head, the skull and sella, and pituitary gland was no abnormal development. When he was 11.5years old, he started using rhGH (4IU qd). Well, at that time of Aug. his height was 144.0cm and weight was 37.3kg. Suggested him to use rhGH (5IU qd).We can see that his treatment has been very effective of height, but his liver B scan showed mild fatty liver. Treatment with lipid metabolism has been unsatisfactory.
